# MDCT-Based Finite Element Analyses: Are Measurements at the Lumbar Spine Associated with the Biomechanical Strength of Functional Spinal Units of Incidental Osteoporotic Fractures along the Thoracolumbar Spine?

**DOI:** 10.3390/diagnostics11030455

**Published:** 2021-03-06

**Authors:** Nico Sollmann, Nithin Manohar Rayudu, Long Yu Yeung, Anjany Sekuboyina, Egon Burian, Michael Dieckmeyer, Maximilian T. Löffler, Benedikt J. Schwaiger, Alexandra S. Gersing, Jan S. Kirschke, Thomas Baum, Karupppasamy Subburaj

**Affiliations:** 1 Department of Diagnostic and Interventional Neuroradiology, School of Medicine, Klinikum rechts der Isar, Technical University of Munich, Ismaninger Str. 22, 81675 Munich, Germany; Nico.Sollmann@tum.de (N.S.); Anjany.Sekuboyina@tum.de (A.S.); Egon.Burian@tum.de (E.B.); Michael.Dieckmeyer@tum.de (M.D.); Maximilian.Loeffler@uniklinik-freiburg.de (M.T.L.); Benedikt.Schwaiger@tum.de (B.J.S.); Jan.Kirschke@tum.de (J.S.K.); Thomas.Baum@tum.de (T.B.); 2 TUM-Neuroimaging Center, Klinikum rechts der Isar, Technical University of Munich, 81675 Munich, Germany; 3 Department of Diagnostic and Interventional Radiology, University Hospital Ulm, Albert-Einstein-Allee 23, 89081 Ulm, Germany; 4 Engineering Product Development (EPD) Pillar, Singapore University of Technology and Design (SUTD), Singapore 487372, Singapore; Rayudu_nithin@mymail.sutd.edu.sg (N.M.R.); Longyu_yeung@mymail.sutd.edu.sg (L.Y.Y.); 5 Department of Diagnostic and Interventional Radiology, University Medical Center Freiburg, Hugstetter Str. 55, 79106 Freiburg im Breisgau, Germany; 6 Institute of Neuroradiology, University Hospital, LMU Munich, Marchioninistrasse 15, 81377 Munich, Germany; Alexandra.Gersing@med.uni-muenchen.de; 7 Changi General Hospital, 2 Simei Street 3, Singapore 529889, Singapore

**Keywords:** bone mineral density, finite element analysis, functional spinal unit, incidental fracture, multi-detector computed tomography, osteoporosis, vertebral fracture

## Abstract

Assessment of osteoporosis-associated fracture risk during clinical routine is based on the evaluation of clinical risk factors and T-scores, as derived from measurements of areal bone mineral density (aBMD). However, these parameters are limited in their ability to identify patients at high fracture risk. Finite element models (FEMs) have shown to improve bone strength prediction beyond aBMD. This study aims to investigate whether FEM measurements at the lumbar spine can predict the biomechanical strength of functional spinal units (FSUs) with incidental osteoporotic vertebral fractures (VFs) along the thoracolumbar spine. Multi-detector computed tomography (MDCT) data of 11 patients (5 females and 6 males, median age: 67 years) who underwent MDCT twice (median interval between baseline and follow-up MDCT: 18 months) and sustained an incidental osteoporotic VF between baseline and follow-up scanning were used. Based on baseline MDCT data, two FSUs consisting of vertebral bodies and intervertebral discs (IVDs) were modeled: one standardly capturing L1-IVD–L2-IVD–L3 (FSU_L1–L3) and one modeling the incidentally fractured vertebral body at the center of the FSU (FSU_F). Furthermore, volumetric BMD (vBMD) derived from MDCT, FEM-based displacement, and FEM-based load of the single vertebrae L1 to L3 were determined. Statistically significant correlations (adjusted for a BMD ratio of fracture/L1–L3 segments) were revealed between the FSU_F and mean load of L1 to L3 (r = 0.814, *p* = 0.004) and the mean vBMD of L1 to L3 (r = 0.745, *p* = 0.013), whereas there was no statistically significant association between the FSU_F and FSU_L1–L3 or between FSU_F and the mean displacement of L1 to L3 (*p* > 0.05). In conclusion, FEM measurements of single vertebrae at the lumbar spine may be able to predict the biomechanical strength of incidentally fractured vertebral segments along the thoracolumbar spine, while FSUs seem to predict only segment-specific fracture risk.

## 1. Introduction

Osteoporosis is characterized by reduced bone mass and microarchitectural bone deterioration, which can result in fragility fractures [1,2,3]. Such fragility fractures are associated with decreases in health-related quality of life and premature mortality of affected patients [4,5,6,7,8]. Further, the presence of the initial fracture is a major risk factor for the occurrence of further fractures over time. In patients who have sustained a fracture, an increased risk of about 86% was reported for another fracture [9]. In this regard, vertebral fractures (VFs) represent one of the most frequent entities of fragility fractures in osteoporosis [1,2,3].

The clinical reference modality for diagnosing osteoporosis and for evaluating fracture risk is dual-energy X-ray absorptiometry (DXA), which enables the measurement of areal bone mineral density (aBMD) [1,10,11]. However, the T-score derived from DXA-based aBMD is inadequate as the only diagnostic criterion for identifying patients at high fracture risk, which is due to overlaps of DXA-derived aBMD values in patients with and without osteoporotic fractures [12,13]. This shortcoming has driven the emergence of alternative techniques, such as computed tomography (CT) as a widely used modality [14,15,16]. Specifically, CT data acquired for other purposes than distinct quantitative evaluation of bone health can be used opportunistically to overcome the limitations of DXA by measuring both volumetric BMD (vBMD) and other parameters beyond, which are essential to understand better the multifarious variables of bone quality [14,15,16].

Based on CT data, finite element analysis (FEA) is an approach that is capable of providing detailed parameters on vertebral bone strength and incident fracture risk prediction in osteoporotic patients [17,18,19]. FEA can relate morphological variation and properties to functional characteristics, thus enabling reductions of complex geometries into a finite number of elements with simple geometries and allowing the strain to be modeled across the surface and throughout the internal architecture of a structure like the vertebral body [20,21,22]. In particular, FEA is considered the gold-standard method to assess vertebral strength and has been repeatedly applied to CT of the spine [17,23,24,25,26,27,28,29]. Specifically, FEA-based vertebral strength measurements have been shown to outperform aBMD in predicting incident VFs [17,23,24]. Furthermore, FEA-based vertebral strength measurements showed strong correlations with actual loads of human cadaveric vertebrae according to in-vitro biomechanical testing [26,27,28]. Using FEA, lumbar and thoracic spine measurements have similar sensitivity and specificity for predicting incident VFs [29].

The common source for FEA-derived vertebral strength measurements has been the single vertebral body in most previous studies [18,30,31]. However, recent research underscores the importance of the functional spinal unit (FSU), which can be defined as at least two adjacent vertebrae with the intervertebral disc (IVD) [25,32]. As such, the FSU should be a more realistic model than the isolated single vertebral body to analyze the actual total load and its distribution [25,32]. Specifically, one recent study demonstrated that FEA-predicted loads of FSUs best predicted experimentally measured loads of FSUs (R^2^ = 0.93, *p* < 0.0001) [25]. Hence, the FEA of single vertebral bodies does most likely not deliver the entire information on actual bone strength and fracture prediction, which could be overcome by incorporating FSUs in an attempt to facilitate a more realistic in-vivo scenario. However, methodologically, FEA is a rather demanding approach, which requires sufficient computational power for accurate modeling. Thus, calculations of FSUs might not yet be performed for the whole spine. Therefore, setting the focus on how FEA of single vertebrae and FSUs at the lumbar spine interrelate, and investigating whether lumbar FEA would be able to predict loads of FSUs at other locations along the spine seems warranted.

Against this background, the present study applied FEA to CT data of the spine in patients who sustained an incidental osteoporotic VF, considering targeted FSUs around the sites of VFs. We investigated whether FEA measurements at the lumbar spine are associated with the biomechanical strength of FSUs with incidental osteoporotic VFs along the thoracolumbar spine.

## 2. Materials and Methods

### 2.1. Setup and Study Cohort

This retrospective study was approved by the local institutional review board (registration number: 27/19 S) and was conducted in accordance with the Declaration of Helsinki. The requirement for written informed consent was waived due to the study’s retrospective design.

A cohort of 11 patients (5 females and 6 males, median age: 67 years, age range: 41–75 years) was included in this study, which was part of a previously reported sample analyzed for different purposes [33]. These patients were retrospectively identified in our hospital-intern digital Picture Archiving and Communication System (PACS). All patients (1) had undergone thoracic and abdominal imaging with the same multi-detector CT (MDCT) system twice (baseline and long-term follow-up) for an oncological indication to rule out tumor recurrence, (2) had a history of cancer (such as esophageal, lung, or colorectal cancer) and chemotherapy, and (3) showed a new incidental osteoporotic VF on follow-up MDCT, which was not present during baseline MDCT. Patients were not considered in the case of any history of bone disease except for osteoporosis (such as hematologic, metastatic, or metabolic disorders affecting bone health).

The presence of VFs was determined in sagittal reformations of the spine by a board-certified radiologist with 11 years of experience, using the classification system described by Genant et al. [34]. The median interval between baseline and follow-up MDCT was 18 months (range: 5–41 months). The site of the incidental osteoporotic VF that occurred between baseline and follow-up scanning affected the following vertebral bodies: T12 (four patients), T9 (two patients), L1 (two patients), T8 (one patient), T11 (one patient), and L3 (one patient).

### 2.2. Computed Tomography Scanning

All baseline and follow-up imaging studies were performed with the same 64-row MDCT scanner (Somatom Sensation Cardiac 64; Siemens Medical Solutions, Erlangen, Germany). A reference phantom (Osteo Phantom; Siemens Medical Solutions, Erlangen, Germany) was placed in the scanner mat beneath the patients during image acquisition for calibration purposes.

The scanning protocol was tailored to oncological needs and acquired during clinical routine, capturing the thorax and abdomen by contrast-enhanced MDCT in the portal venous phase. The scanning parameters included a tube voltage of 120 kVp, an adapted tube load of average 200 mAs, and a minimum collimation of 0.6 mm. Raw image data acquired in the axial plane were standardly reformatted to obtain sagittal images of the spine with a slice thickness of 3 mm and a standard bone kernel. Intravenous administration of a contrast medium (Imeron 400; Bracco, Konstanz, Germany) was performed with a high-pressure injector (Fresenius Pilot C; Fresenius Kabi, Bad Homburg, Germany) using a delay of 70 s, a flow rate of 3 mL/s, and an individual weight-dependent dose (80 mL for body weight up to 80 kg, 90 mL for body weight up to 100 kg, and 100 mL for body weight over 100 kg). Besides, all patients were given 1000 mL of an oral contrast medium (Barilux Scan; Sanochemia Diagnostics, Neuss, Germany).

### 2.3. Calculation of Bone Mineral Density

Vertebral vBMD assessment was performed using baseline images of the vertebral bodies from L1 to L3, the later incidentally fractured vertebral body, and its adjacent upper and lower vertebrae. The most central slice depicting the vertebral body was selected from the sagittal reformations. Manual regions of interest (ROIs) were placed equidistant to both endplates in the trabecular compartment of the anterior vertebral body, which was followed by extraction of Hounsfield Units (HU) per vertebral body and patient [35,36]. The ROI diameters enclosed approximately two-thirds of the height of the vertebral bodies.

Using the phantom (two phases, water-like and bone-like compartment with values of 0 and 200 mg/mL hydroxyapatite (HA)), vBMD values were calculated based on the following HU–BMD calibration equation [35,36]: (BMD)MDCT = (HAb/(HUb − HUw)) × (HU − HUw). HUw and HUb represent the attenuation values (in HU) of the phantom’s water and bone components, respectively, and HAw and HAb represent the HA values of the two components, respectively. The derived values were converted to standard values of quantitative CT (QCT) using the following linear relation [35,36]: (BMD)QCT = 0.69 × (BMD)MDCT − 11 mg/mL.

### 2.4. Vertebral Body and Intervertebral Disc Segmentations

The vertebral bodies from T5 to L5, including the posterior elements, were automatically segmented from the MDCT images using a deep-learning-driven framework (https://anduin.bonescreen.de accessed on 6 March 2021). This algorithm identifies the spine, labels each vertebral body, and creates corresponding segmentation masks [37,38]. In addition, manual segmentations of the IVDs were generated by a board-certified radiologist with 11 years of experience, using the open source software Medical Imaging Interaction Toolkit (MITK; developed by the Division of Medical and Biological Informatics, German Cancer Research Center, Heidelberg, Germany; www.mitk.org accessed on 6 March 2021). Adjacent soft tissue or other surrounding structures were spared.

### 2.5. Finite Element Modeling and Analysis

The MDCT scan data and the segmentation masks of T5 to L5 and IVDs were imported to the commercial three-dimensional (3D) medical image processing software Mimics (Materialise NV, Harislee, Belgium), and 3D vertebral models and IVD models were generated. These 3D models were then imported to 3-Matic software (Materialise NV, Harislee, Belgium) for meshing.

Tetrahedral elements (C3D4 in the ABAQUS material library) were used to mesh the geometry and capture the bone contour for downstream analysis. The bone’s non-homogeneous and non-isotropic material behavior was captured by considering image intensity-based material-mapping relations. The IVDs were also meshed with tetrahedral elements for accurate representation. Table 1 shows the HU–density–elasticity material-mapping relations and IVD material properties used in the current study.

**Figure 1:**
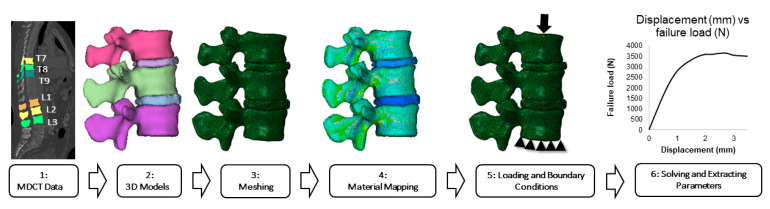
Overview of the analysis methodology for finite element models (FEMs). This figure provides an overview of the six-step analysis approach for the generation of FEMs, starting with extraction of multi-detector computed tomography (MDCT) datasets and resulting in solving and extracting parameters.

For FSU models, the meshed and material-mapped 3D vertebrae models were then imported to the commercial analysis software ABAQUS (version 6.10; Dassault Systèmes, Johnston, RI, USA) for downstream FEA. Then, the vertebrae and IVDs were assembled and FSUs were developed. Tie constraints were applied between the vertebrae and IVDs and also between disc and annulus, and a no-penetration-contact condition was applied to the posterior elements. Then, loading and boundary conditions were applied and the model was solved. In the current study, the compression loading condition was simulated by constraining all the nodes on the inferior surface of the lower vertebrae, and displacement loading was applied on the superior surface of the top vertebrae (Figure 1). 

Then, the finite element model (FEM) of the FSUs centered on the level of the incidental osteoporotic VF (defined as FSU_F) and the FEM of the FSU including L1 to L3 (defined as FSU_L1–L3) were calculated. The peak of the load–displacement curve was considered as failure load, and the corresponding displacement for the load was considered as failure displacement. The FEA methodology used in the current study has been validated experimentally in previous studies [18,25,30,31].

For single vertebral models, the meshed and material-mapped L1, L2, and L3 vertebral models were imported to the analysis software ABAQUS (version 6.10; Dassault Systèmes, Johnston, RI, USA). In the current study, the compression loading condition was simulated by fixing the inferior surface of the vertebrae, and normal displacement loads were applied on the superior surface. The failure load and displacement were extracted from the load–displacement curve, and these values were used for further statistical analyses. The single vertebral analysis methodology used in the current study has been validated experimentally in previous studies [31,33].

Figure 1 shows the six-step analysis methodology followed in the current study for the analysis of FSU models. For single vertebral model simulations, a similar six-step methodology was used, and data analysis was performed on the extracted parameters [31,33].

### 2.6. Statistical Analysis

SPSS (version 26.0; IBM SPSS Statistics for Windows, IBM Corp., Armonk, NY, USA) and GraphPad Prism (version 6.0; GraphPad Software Inc., San Diego, CA, USA) were used for statistical data analyses and the generation of graphs of a correlation matrix. A *p*-value of < 0.05 was considered statistically significant.

The vBMD of the vertebrae L1 to L3, the vBMD of the incidentally fractured vertebral body and its adjacent upper and lower vertebrae, the values of displacement from L1 to L3, and the load of L1 to L3 were averaged across the three respective vertebral bodies. Descriptive statistics including mean ± standard deviation (SD) or median with ranges or absolute frequencies were calculated for cohort demographics as well as the FEMs (the FEM of the FSU centered on the level of the incidental osteoporotic VF and the FEM of the FSU including L1 to L3), vBMD, displacement, and load of L1 to L3. Furthermore, the ratio of the mean vBMD of the incidentally fractured vertebral body and its adjacent upper and lower vertebrae to the mean vBMD of the vertebrae L1 to L3 was calculated. This was done to take the vBMD heterogeneity along the spine into account, as suggested previously [46]. Partial correlation analyses adjusting for this ratio were performed between the FEM of the FSU centered on the level of the incidental osteoporotic VF as the dependent variable and the FEM of the FSU including L1 to L3, vBMD, displacement, and load averaged over L1 to L3 as the independent variables.

## 3. Results

**Figure 2:**
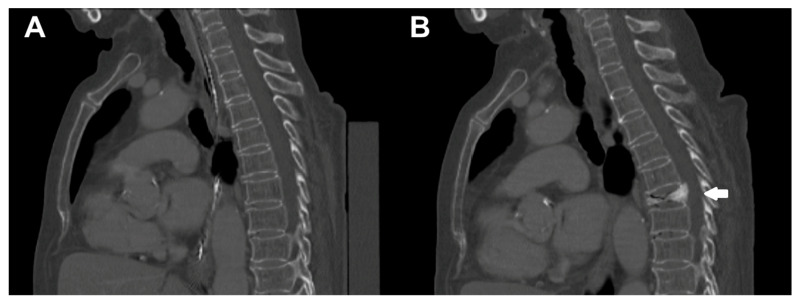
Illustrative patient case. This figure depicts sagittal reformations of the spine of a 78-year-old female patient (**A**) at baseline and (**B**) during follow-up imaging by multi-detector computed tomography (MDCT). During the interval of 8 months between baseline and follow-up scanning, the patient sustained an incidental osteoporotic vertebral fracture (VF) of T7 (*white arrow*).

Extraction of vBMD as well as calculation of FEMs, load, and displacement were possible for all enrolled patients. Table 2 shows the mean values and ranges of these parameters for the whole cohort. Figure 2 depicts an illustrative patient case.

**Figure 3:**
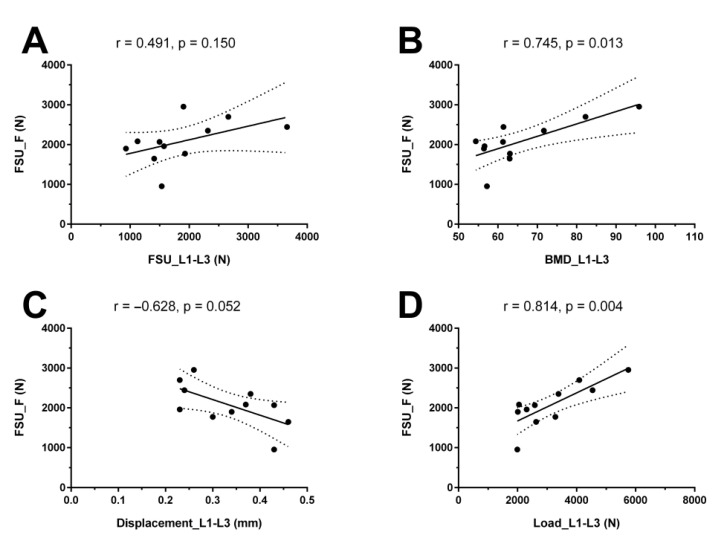
Correlation analyses with finite element models (FEMs). This graph depicts the correlation of the FEM of the functional spinal unit (FSU) centered at the level of the incidental osteoporotic vertebral fracture (VF) as the dependent variable (*y-axis*, FSU_F) with the (**A**) FEM of the FSU including vertebrae L1 to L3 (FSU_L1–L3), (**B**) vBMD of L1 to L3 (BMD_L1–L3), (**C**) displacement of L1 to L3 (Displacement_L1–L3), and (**D**) load of L1 to L3 (Load_L1–L3). Individual values are given as single dots, together with the adjusted correlation coefficient r and related *p*-values of respective correlation analyses.

There was no statistically significant correlation between the FSU_F and FSU_L1–L3 (r = 0.491, *p* = 0.150) or between the FSU_F and the mean displacement of L1–L3 (r = −0.628, *p* = 0.052; Figure 3). In contrast, correlation analyses revealed a statistically significant correlation (adjusted for the BMD ratio of fracture/L1–L3 segments) between the FSU_F and vBMD of L1–L3 (r = 0.745, *p* = 0.013) as well as between the FSU_F and mean load of L1–L3 (r = 0.814, *p* = 0.004; Figure 3).

## 4. Discussion

This study used the FEA of FSUs in patients who sustained an incidental osteoporotic VF in order to investigate whether FEA parameters of the lumbar spine can predict incidental fractures in any other segments of the thoracolumbar spine. We observed associations between the FSU_F and mean vBMD of L1–L3 and between the FSU_F and mean load of L1–L3.

Several previous studies have demonstrated that FEA-based vertebral strength measurements are associated with the actual vertebral load derived from in-vitro mechanical testing using cadaveric vertebrae [23,27,28]. This finding is in agreement with the present study, showing correlations between FEMs and the vertebral load. Bone is a complex, anisotropic, and non-homogeneous material. If a bone is going to fail in the future, it will become weaker in advance, implying that load and displacement values will be altered. However, the present study extends beyond previous analyses by incorporating the FSU, consistent with recent work that suggests the relevance of also considering IVDs in an attempt to simulate a more realistic setting [25,32]. In this context, one recent study has already demonstrated that the FEA-predicted load of FSUs best predicted the experimentally measured load of FSUs [25]. Specifically, in this previous study, the role of FSUs was evaluated by comparing the FEA-predicted load with the experimentally measured failure load, and by comparing this correlation with that of FEA-predicted failure load and BMD of the single central vertebra with the experimentally measured load [25]. Importantly, the present work employed the same, thus experimentally validated methodology to model and analyze FSUs from routine clinical MDCT data. Further, in the present study, we investigated whether FEA measurements at the thoracolumbar spine are associated with the biomechanical strength of FSUs with incidental osteoporotic VFs. This research question has not been addressed in the literature. Of note, most of the previous work focused on analyzing only the lumbar spine to predict fractures in any section of the spine, which may not necessarily represent an adequate surrogate.

In this study, the lack of a significant correlation between the FEM of the FSU centered on the level of the incidental osteoporotic VF and the FEM of the FSU including L1 to L3 may highlight the importance of considering the site of VFs. While the lumbar spine is the reference site for conventional BMD measurements via DXA or QCT, it may not be enough to standardly consider lumbar vertebrae as a surrogate for the whole spine. However, recent work using FEA suggests that both CT-based bone density and strength measurements from the thoracic and lumbar spine may be used to identify individuals at high risk for VFs [29]. Specifically, bone measurements from T8 and L2 predicted incident VFs equally well, regardless of fracture location, and lumbar and thoracic spine bone measurements had similar sensitivity and specificity for predicting incident VFs [29]. Yet, the FSU should refine results as the vertebral body is analyzed in conjunction with biomechanically relevant, connected surrounding structures, which can impact bone strength and force distributions and are particularly crucial in osteoporosis where the stability of weakened bone could specifically depend on its stabilizing surrounding structures, including IVDs. However, the time needed for FEA depends on the computational resources available, with conventionally used clinical setups not yet fulfilling the requirements for timely completion of required analyses steps (in our case, a 32 GB RAM and 8 cores were used to run FEA, with a pre-processing time of ~300 min and a solving time of ~360 min). With the introduction of hardware with higher computational power in clinically used systems, FEA may become feasible more broadly in the near future and could potentially be incorporated in the assessment of osteoporosis in a routine clinical scenario.

For FEA, existing CT images from routine clinical scans were used in this study. The opportunistic use of such CT data is relevant for minimizing the radiation dose applied to the patient, with ionizing radiation due to CT imaging showing drastic rises over the recent years [47,48]. If existing data can be used to assess musculoskeletal characteristics alongside initial clinical indications for imaging (e.g., oncological staging for the present study), additional CT or DXA that would increase the patients’ radiation exposure could be spared. Specifically, in patients with cancer, pathological conditions like sarcopenia, cachexia, and osteoporosis are frequently observed [49,50,51]. Therefore, this group may particularly benefit from the opportunistic use of CT data, potentially avoiding additional scanning for dedicated osteoporosis screening. Of note, FEA does not require CT data with elevated exposure; instead, a dose reduction of at least 75% compared to standard scanning is still feasible for adequate prediction of the in-vivo vertebral load [18]. Similarly, dose reduction in the range of 80 to 500 mA has no significant impact on the FEA-predicted vertebral load, as shown by an ex-vivo setup using thoracic mid-vertebrae specimens [19].

The following limitations need to be considered. First, the small cohort size represents a shortcoming, which indicates that the results of this study should be followed up in larger samples. Yet, this study was motivated by methodological advances with FEA of FSUs in the center of analyses, rendering assessment in larger clinical cohorts premature. Based on the results of the present study, future work may implement the algorithms and analysis steps in representative clinical cohorts. Second, this study was performed in oncological patients; thus, the generalizability of findings and transfer to patients with other diseases or healthy controls has to be investigated in the future. Third, we used MDCT data that were acquired after administration of an intravenous contrast agent. Thus, results from non-contrast MDCT may differ due to the effects of contrast media on obtained measurements. Yet, the potential impact of contrast medium accumulation in the vertebral venous plexus on measurements was reduced by the approach of ROI placement, which has also been similarly achieved in previous work that extracted vBMD from routine contrast-enhanced MDCT scans [35,36]. Furthermore, using a validated conversion equation for the used scanner, the influence of the intravenous contrast agent was adjusted [35]. The conversion showed high reproducibility, as indicated by a short-term and long-term reproducibility error of 2.09% and 7.70%, respectively [36]. Fourth, the FSU considered in this study was composed of adjacent vertebrae with IVDs, yet the anatomo-functional conditions at the spine are more complex and also involve structures like ligaments and facet joints. Further work is needed to also precisely include such structures in biomechanical modeling at the spine. Fifth, the time to follow-up scanning showed variability between patients, which is due to differences in the time point selection for scanning during the oncological staging of the included subjects. While this resembles a clinical reality and may be inherent to the opportunistic use of existing imaging data, future studies may aim to select patients with more homogeneous inter-scan intervals to exclude bias in measurements due to differences related to the time point of image acquisitions.

## 5. Conclusions

This study provides evidence of the relevance of FEA derived from MDCT for assessment of the biomechanical characteristics of the spine using FSUs. Specifically, the FEM of the FSU centered at the level of the incidental osteoporotic VF was associated with the mean vBMD and mean load of L1 to L3. In contrast, it was not significantly correlated with displacement or the FEM of L1 to L3. Hence, FEMs of single vertebrae may predict the biomechanical strength of incidentally fractured vertebral segments along the thoracolumbar spine, while FSUs seem to predict only the segment-specific fracture risk. Future studies should follow up on these findings to further explore the need for incorporating FSUs in modeling of the spine, with the aim to provide realistic scenarios of vertebral bone strength and fracture prediction. Such studies are also needed to further investigate the potential effects of resolution and contrast agents on dedicated FEA, ideally combining in-vivo data with additional experimental confirmation. In this regard, FEA based on MDCT has the potential to become a valuable adjunct methodology for routinely performed vBMD measurements in the future.

## Figures and Tables

**Table 1:** Property and mapping relations.

Property	Mapping Relations
**Vertebral material properties**	
Apparent density (ρ_app_ in kg/m^3^) [39]	ρ_app_ = 47 + 1.122 * HU
Ash density (ρ_ash_ in kg/m^3^) [40]	ρ_ash_ = 0.6 * ρ_app_
Elastic modulus (E in MPa) [39]	E_z_ = −349 + 5.82 * ρ_app_ E_x_ = E_y_ = 0.333 E_z_ Z-axial direction of the vertebra
Shear modulus (G in MPa) [26]	G_xy_ = 0.121 E_z_ G_xz_ = G_yz_ = 0.157 E_z_
Poisson ratio (V) [26]	Vxy = 0.381 Vxz = Vyz = 0.104
Maximum principal stress limit (σ in MPa) [41]	σ = 137 * ρ_ash_^1.88^, ρ_ash_ < 0.317 σ = 114 * ρ_ash_^1.72^, ρ_ash_ > 0.317
Plastic strain (ε_AB_) [42]	ε_AB_ = −0.00315 + 0.0728 ρ_ash_
Minimum principal stress limit (σ_min_ in MPa) [42]	σ_min_ = 65.1 * ρ_ash_^1.93^
**Intervertebral disc properties**	
***Annulus***	
Elastic modulus (E in MPa) [43]	E = 500
Poisson ratio (V) [43]	0.3
***Nucleus***	
Elastic modulus (E in MPa) [44]	E = 1
Poisson ratio (V) [45]	0.475

This table depicts patient-specific image-intensity-based mapping relations (density (ρ), intensity (in Hounsfield Units [HU], modulus (E)) used in the current computational study for the modeling of realistic bone anisotropic material behavior.

**Table 2:** Results of finite element analysis (FEA) and volumetric bone mineral density (vBMD) measurements.

Item	Mean ± SD	Range
FSU_F (N)	2075.42 ± 518.11	952.35–2954.57
FSU_L1–L3 (N)	1867.30 ± 740.07	928.14–3657.75
BMD_L1–L3 (mg/mL)	65.75 ± 12.21	54.38–95.88
Displacement_L1–L3 (mm)	0.34 ± 0.08	0.23–0.46
Load_L1–L3 (N)	3147.24 ± 1161.40	1991.94–5752.74

This table shows the mean ± standard deviation (SD) and range for the finite element model (FEM) of the functional spinal unit (FSU) centered at the level of the incidental osteoporotic vertebral fracture (VF) and the FEM of the FSU including L1 to L3 (FSU_F and FSU_L1–L3, in N). Furthermore, it displays respective values for the vBMD of the vertebrae L1 to L3 (BMD_L1–L3, in mg/mL), displacement of L1 to L3 (Displacement_L1–L3, in mm), and load of L1 to L3 (Load_L1–L3, in N).

## Data Availability

The raw data supporting the conclusions of this article will be made available by the authors, without undue reservation.

## Abbreviations

3D	Three-dimensional
aBMD	Areal bone mineral density
CT	Computed tomography
DXA	Dual-energy X-ray absorptiometry
FEA	Finite element analysis
FEM	Finite element model
FSU	Functional spinal unit
HA	Hydroxyapatite
fHU	Hounsfield Units
IVD	Intervertebral disc
MDCT	Multi-detector computed tomography
PACS	Picture Archiving and Communication System
QCT	Quantitative computed tomography
ROI	Region of interest
SD	Standard deviation
vBMD	Volumetric bone mineral density
VF	Vertebral fracture
